# Outcomes of Hospitalized Patients With Fecal Occult Positive Stool Prior to Cardiac Catheterization in Acute Coronary Syndrome (ACS)

**DOI:** 10.7759/cureus.34263

**Published:** 2023-01-27

**Authors:** Lauren Searls, Frank H Annie, Julton Tomanguillo, James R Campbell, Suzanne Kemper, Vishnu Vardhan Reddy Naravadi

**Affiliations:** 1 Internal Medicine, West Virginia University (WVU) Medicine Internal Medicine Residency – Charleston, Charleston Area Medical Center, Charleston, USA; 2 Cardiology, Charleston Area Medical Center, Charleston, USA; 3 Internal Medicine, West Virginia University (WVU) Medicine Internal Medicine Residency – Charleston Area Medical Center, Charleston, USA; 4 Internal Medicine, Charleston Area Medical Center Institute for Academic Medicine, Charleston, USA; 5 Internal Medicine, West Virginia University School of Medicine Charleston Division, Charleston, USA; 6 Outcomes Research, Charleston Area Medical Center (CAMC) Health Education and Research Institute, Charleston, USA; 7 Internal Medicine/Gastroenterology, West Virginia University (WVU) Physicians of Charleston, Charleston, USA; 8 Gastroenterology, Charleston Area Medical Center, Charleston, USA

**Keywords:** patients, hospitalized, acs, fobt, nstemi

## Abstract

Introduction

Cardiac catheterization is an essential component of patient care in Acute Coronary Syndrome (ACS). Fecal occult blood testing (FOBT) has been used in the inpatient setting to evaluate the risk of bleeding with dual anti-platelet therapy prior to cardiac catheterization although no guidelines exist for this indication and FOBT testing in the inpatient setting is not recommended for evaluation of GI blood loss. We sought to assess the outcomes of patients with fecal occult positive stool prior to cardiac catheterization compared to those that did not undergo FOBT during admission for non-ST-elevation myocardial infarction (NSTEMI).

Methods

We identified patients between 18 and 90 years old with admission for NSTEMI in the Trinetx Research Network from January 1, 2019 to December 31, 2020. Patients were then divided into those who had an FOBT prior to cardiac catheterization and those that did not have an FOBT. We compared all-cause mortality, bleeding, troponin levels, and length of stay between propensity-matched (PSM) pairs of patients.

Results

We identified 46,349 that met inclusion criteria, of which 1,728 had an FOBT (3.7%) and 44,621 (96.3%) had no FOBT prior to cardiac catheterization. Patients in the FOBT group were older and had a higher prevalence of hypertension, coronary artery disease, heart failure, diabetes, chronic obstructive pulmonary disease, and higher BMI. Two well-matched groups of n=1,728/1,728 were used for comparing outcomes. The FOBT group had similar 30-day mortality (4.45% vs 4.01, P=0.56) as well as similar bleeding events (0.98% vs 0.69%, P=0.35). Troponin levels in the FOBT group were on average lower (0.41 vs 0.95, P=0.04). The FOBT groups also had a similar average length of stay of (14.1 days vs 14.2 days, P=0.42). 233 patients who received FOBT underwent endoscopic evaluation with either upper endoscopy or colonoscopy (13.5%), and there was no significant difference in 30-day mortality (6.86% vs 4.7%, P=0.321). Among patients who underwent endoscopy, 72 had some form of endoscopic intervention (30.9%). There was no difference in 30-day mortality between patients undergoing endoscopy with intervention and without intervention (14.49%/14.49%) P=1.00. Readmission was similar between patients undergoing endoscopy with and without intervention.

Conclusions

In a large multi-center national database, we observed similar outcomes in patients who were admitted with NSTEMI and had FOBT and those not receiving FOBT in terms of all-cause mortality and bleeding events. In patients with positive FOBT, endoscopy with and without intervention we observed no significant difference in 30-day mortality. We conclude that there is no compelling evidence for FOBT testing in patients with NSTEMI.

## Introduction

Cardiac catheterization is an essential component in the treatment of acute coronary syndrome (ACS). Stent placement is a significant risk factor for new gastrointestinal bleeding, with a risk between 1.3% and 2.4% for GI bleeding within 30 days of ACS in patients on dual antiplatelet therapy (DAPT) [1,2]. Gastrointestinal bleeding following ACS is associated with increased morbidity and mortality [3-8]. Fecal occult blood testing (FOBT) has been used in studies in an attempt to predict the need for DAPT discontinuation and assess the bleeding risk of DAPT [9,10]. In these studies, up to 25% of patients had at least one positive FOBT, and >80% of patients with positive FOBT successfully remained on DAPT. FOBT is recommended as a non-invasive colon cancer screening test, however, is commonly ordered in the inpatient setting for anemia and suspected gastrointestinal bleeding [11,12]. Inpatient FOBT has been highlighted in The Society of Hospital Medicine’s choosing wisely campaign and is problematic secondary to high type 1 error (around 50%) [13]. The timing and safety of endoscopy in patients with ACS and overt gastrointestinal bleeding have been studied. Urgent endoscopy has been shown to be beneficial prior to cardiac catheterization in patients with upper GI blood loss, hemodynamic instability, or hematemesis. In patients with less severe clinical features, endoscopy was safely delayed until after cardiac catheterization [3,14]. Little data exists regarding performing endoscopy prior to cardiac catheterization in patients with non-ST-elevation myocardial infarction (NSTEMI) on the basis of positive FOBT. The aim of this study is to assess the utility of FOBT in patients hospitalized with NSTEMI and to assess the utility of endoscopy on the basis of positive FOBT.

## Materials and methods

We identified adult patients aged 18-90 years that had an inpatient admission for NSTEMI between January 1, 2019 to December 31, 2020 using the TriNetX research network database, which comprises 57 healthcare organizations. We identified (n=46,349) patients meeting inclusion criteria, and divided patients into cohorts with FOBT during admission (n=1,728) compared to those who did not undergo FOBT (n=44,621). In order to understand potential differences in the groups, we constructed a 1:1 propensity match model to control for the literature-driven covariates which included age, white, male, female, black or African American, Hypertension, atherosclerotic heart disease of the native coronary artery, chronic heart failure, diabetes mellitus, chronic obstructive pulmonary disease, BMI< 30 (Tables 1, 2).

**Table 1:** List of codes used to identify the study cohort claim evidence

ICD - CPT Codes From Trinetx Platform
G0328, Colorectal cancer screening fecal occult blood test
82274, Blood occult, by fecal hemoglobin determination by immunoassay, qualitative feces, 1-3 simultaneous determinations
82956-4, Hedis 2017-2020 value set – FIT-DNA
74243-7, Hedis 2014-2016 value set – FOBT
82959-8, Hedis 2017-2020 value set – FOBT
29771-3, Hemoglobin gastrointestinal lower presence in stool by immunoassay
57803-9, Occult blood panel – stool by immunoassay
2335-8, Hemoglobin gastrointestinal presence in stool
93462 Left heart catheterization by transeptal
93452, Left heart catheterization
931531, combined right heart catheterization and retrograde left heart cauterization
4A023N7, Measurement of Cardiac Sampling
I21.4, Non-St elevation (NSTEMI) myocardial infarction
I22.2, Subsequent non-ST elevation (NSTEMI) myocardial infarction

**Table 2:** Baseline characteristics (PSM Match) SD-standard deviation, COPD-chronic obstructive pulmonary disease, CAD-coronary artery disease, CHF- Congestive heart failure, N-number, * Obesity was defined as a body mass index ³ 30 kg/m2

Baseline Characteristic	Unmatched Cohorts				Propensity Matched Cohorts			
	FOBT NSTEMI (N=1,728)	No-FOBT NSTEMI (N=44,621)	P-Value	Standardized Mean Difference	FOBT NSTEMI (N=1,728)	No-FOBT NSTEMI (N=1,728)	P-value	Standardized Mean Difference
Age at Index	67.8±11.5	64.2± 12.6 69.68%	<0.01	0.30	67.8±11.5	67.1±11	0.41	0.03
White	75.93%	75.08%	0.423	0.02	75.93%	77.78%	0.20	0.04
Male	56.19%	63.24%	<0.01	0.14	56.19%	57.47%	0.45	0.03
Female	42.82%	36.09%	<0.01	0.14	42.82%	41.90%	0.58	0.02
Black	14.87%	13.53%	0.11	0.04	14.87%	14.24%	0.60	0.02
Hypertensive diseases	92.54%	66.06%	<0.01	0.70	92.54%	93.00%	0.60	0.02
CAD	84.43%	49.02%	<0.01	0.81	84.43%	85.24%	0.51	0.02
CHF	63.48%	29.89%	<0.01	0.71	63.48%	62.91%	0.72	0.01
Diabetes	58.04%	35.24%	<0.01	0.47	58.04%	58.97%	0.58	0.02
COPD	32.18%	15.98%	<0.01	0.39	32.18%	32.29%	0.94	0.01
BMI ³ 30 kg/m2	71.12%	41.44%	<0.01	0.63	71.12%	69.68%	0.35	0.03

Data source

The Trinetx Inc. (Cambridge, MA) database is a global federal research network that combines real-time data from electronic medical records into a user-friendly platform for easy user access.

Study sample 

We queried the Trinetx (Research Network) which is a collection of 57 healthcare organizations from January 1, 2019 to December 31, 2020. We identified (n=46,349) aged 18-90. Trinetx, LLC is compliant with (HIPPA) and US federal law which protects the privacy and security of health care data. 

Statistical analyses

The TriNetX platform uses descriptive statistics and creates several frequencies with differing percentages that are transferred into categorical variables using standard mean ± deviation for continuous measures. In order to understand baseline characteristics, Pearson’s chi-squared test is created for categorical variables. To account for potential differences in the cohorts a 1:1 propensity match using logistic regression to create two well-matched cohorts for analysis. The propensity analysis uses logistic regression for scores for differing propensity metrics for differing selected covariates. The propensity score match uses the Python libraries (NumPy and Sklearn). The final results compare the results to R to compare and verify the results. A final step in the verification process uses the nearest neighbor function set to a tolerance level of 0.01 and a deference of value >0.1. To address the endpoint of mortality a measure of difference of association was used and well as a Kaplan Meier as a verification test. 

Sensitivity analysis

In order to understand any potential external variables that could be affecting the design of the study. a falsification endpoint of bleeding was created.

## Results

**Figure 1:**
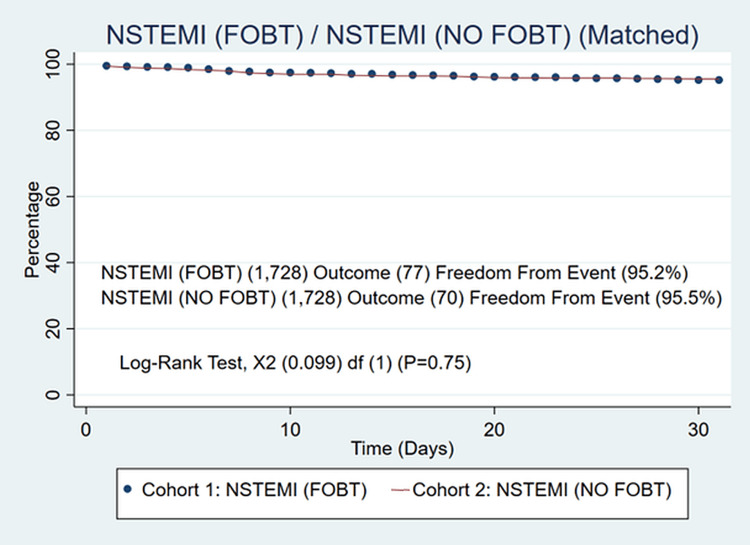
Freedom from all-cause mortality in patients receiving FOBT and not receiving FOBT over time

**Figure 2:**
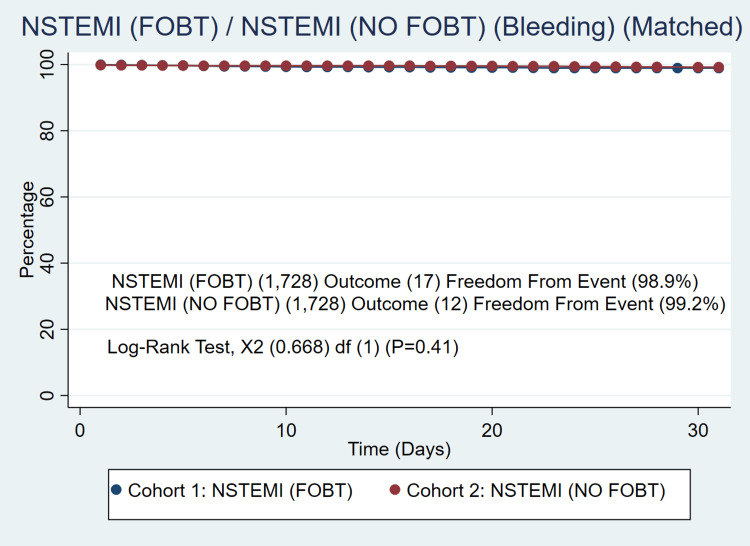
Freedom from bleeding events in patients receiving FOBT and not receiving FOBT over time

We identified 46,349 patients meeting the inclusion criteria. Of those 1,728 had an FOBT administered (3.7%) and 44,621 (96.3%) had no FOBT administered prior to cardiac catheterization. Patients in the FOBT group were older (67.8 ± 11.5 vs 64.2 ± 12.6, P<0.001). The FOBT group also had a higher prevalence of hypertension (92.5% vs 66.1%, P<0.01), coronary artery disease (84.4% vs 49.0%, P<0.01), heart failure (63.5% vs 30.0%, P<0.01), diabetes (58.0% vs 35.2%, P<0.01), chronic obstructive pulmonary disease (32.2% vs 16.0%, P<0.01) and higher BMI (28.8 ± 6.87 vs 29.7 ± 6.87, P<0.001). We were able to create two well-matched groups of n=1,728/1,728. The FOBT group had similar 30-day mortality (4.45% vs 4.01, P=0.56) confirmed with a Kaplan Meier curve in Figure 1 with a log-rank of (P=0.75) as well as similar bleeding events (0.98% vs 0.69%, P=0.35) confirmed with a Kaplan Meier curve in Figure 2 with a log-rank of (P=0.41). Compared to the non-FOBT group, hemoglobin levels in the FOBT group were lower (10.7±2.56 vs 13.1±2.33, P<0.01) and troponin levels were on average lower (0.41 vs 0.95, P=0.04). The FOBT and non-FOBT groups had a similar average length of stay (14.1 days vs 14.2 days, P=0.42). Two hundred and thirty-three patients who received FOBT underwent endoscopic evaluation with either upper endoscopy or colonoscopy during index admission (13.5%). Of patients receiving an endoscopic evaluation, there was no significant difference in mortality (6.86% vs 4.7%, P=0.321). Among patients who underwent endoscopy, 72 had some form of endoscopic intervention (30.9%). There was no difference in 30-day mortality between patients undergoing endoscopy with the intervention compared to those without intervention (14.49%/14.49%) P=1.00 confirmed with log-rank test (92.60% vs 92.37%) P=0.91. Readmission was similar between patients undergoing endoscopy with and without intervention.

## Discussion

Performing endoscopy on patients with ACS is an area of concern for gastroenterologists. Sedation associated with endoscopy is a known stressor on the heart, however, has been shown to be safe following acute myocardial infarction in several studies [3,15-22]. In the absence of upper GI bleeding, hematemesis, or hemodynamic instability, urgent endoscopy can safely be delayed [23]. ACS with stent placement is a significant risk factor for new gastrointestinal bleeding. Patients undergoing standard medical therapy for ACS with DAPT, and low molecular weight heparin are at risk of clinically significant GI bleeding, with 1.3%-2.4% risk of GIB within 30 days of ACS [15,16]. At one year following PCI, GIB is the most common source of bleeding [24]. Gastrointestinal bleeding following an acute cardiovascular event is associated with increased morbidity, and patients undergoing endoscopy who were admitted with ACS are more critically ill in general than those admitted with ACS not undergoing endoscopy. These patients have higher mortality [3-8] increased length of hospital stay [7,8], increased risk of an in-hospital major adverse cardiac event [17], increased blood/platelet infusions [18] as well as increased resource utilization and cost of care than patients without GIB following PCI [25].

FOBT is frequently utilized in inpatient settings for inappropriate reasons [11,26-29]. FOBT (either guaiac-based testing or immunohistochemical) is recommended as a non-invasive screening tool for colorectal cancer, however, is frequently used in the inpatient setting for alternative indications. Anemia and suspected gastroenterology bleeding have been identified as leading factors for an FOBT order [11,29]. A single-center study showed 74% of FOBT tests ordered for anemia were negative, and hospitalized patients with a positive FOBT are more likely to undergo endoscopic procedures during their stay and were more likely to receive a gastroenterology consult [11,29]. The Society of Hospital Medicine has identified inpatient FOBT testing as a quality issue in their Choosing Wisely campaign. Inpatient FOBT testing is problematic due to the high type 1 error (around 50%), and other factors attributing to positive test results. Bleeding from any source, inflammation such as gastritis, certain foods, and medications can lead to false positive results.

Although only clinically indicated as a cancer screening tool and not indicated for inpatient use [28,29]. FOBT testing has also been attempted to predict DAPT discontinuation and bleeding risk post-PCI. Data on using FOBT prior to cardiac catheterization is limited and has been studied regarding the safety profile for DAPT in patients prior to PCI, avoiding premature discontinuation of DAPT in patients receiving coronary stenting, and as an indicator for DAPT discontinuation following PCI. In a study from Japan [9], FOBT was used as a screening tool prior to PCI, and endoscopy was performed on patients with two positive FOBT results. Twenty-five percent of 647 patients screened had one positive FOBT, and 11% tested positive twice. In an additional single-center study [10], patients were screened with endoscopy if FOBT testing was positive prior to cardiac catheterization. FOBT was positive in about 6% of patients and was associated with increased DAPT discontinuation; however, >80% of patients with positive FOBT results successfully remained on DAPT following PCI. Although little data exists regarding endoscopy or FOBT prior to cardiac catheterization in patients with NSTEMI, it is frequently performed in the inpatient setting. Our results show no benefit in performing FOBT prior to cardiac catheterization in patients hospitalized with NSTEMI.

Utilizing a national database allowed for a large sample size including 46,349 patients across multiple health systems. The increased comorbidity noted in the population receiving FOBT is unsurprising, as many of these comorbidities also have an increased risk of gastrointestinal bleeding, especially age. Hemoglobin levels in the group receiving FOBT were significantly lower, suggesting FOBT is ordered as a means to assess for GI blood loss. Lower troponin levels in those receiving FOBT suggest that patients receiving FOBT could have less urgent cardiac risk. Overall, the percentage of patients presenting with NSTEMI and undergoing cardiac catheterization who received FOBT remains low at 3.7%. Our results show no statistically significant difference in 30-day mortality and bleeding events in patients undergoing FOBT. Of patients receiving FOBT, endoscopy was performed in 13.5%, with a slight increase in mortality noted for those patients undergoing endoscopy (6.86% vs 4.7%); however, statistical significance was not noted. 30.9% of patients who underwent endoscopy did have an intervention, however, intervention during endoscopy did not affect mortality in these patients. Skewing of mortality and bleeding events in the population receiving FOBT may correlate with increased overall comorbid conditions noted in patients receiving this testing. Of note, performing FOBT did not increase the hospital's length of stay. Without a difference in mortality or clinically significant GI bleeding, there is no compelling evidence to suggest FOBT prior cardiac catheterization in patients hospitalized with NSTEMI to be beneficial.

Our study was not without limitations. Utilizing a large national database with de-identified data, we were unable to assess if overt bleeding was present on admission. Our study is a retrospective chart review, having the inherited limitations of selection bias and inability to assess incidence. In the future, further assessment is needed to evaluate if endoscopy had a significant impact on the length of stay. Assessment is also needed to evaluate if patients receiving FOBT or undergoing endoscopy had a delay in time to cardiac catheterization compared to patients not receiving FOBT. As this study is a retrospective review utilizing a database, there may have been additional factors such as the need for blood transfusion, development of shock, socioeconomic status, and another clinical acumen that could not be taken into consideration for ordering FOBT. Our study did not differentiate between guaiac-based FOBT (gFOBT) and immunohistochemical FOBT (iFOBT). Hospitalization cost is an important factor that can be assessed in the future, to see if a difference in total hospitalization cost exists in patients receiving FOBT or endoscopy inpatient as a pre-PCI screening.

## Conclusions

We present a large data registry that illustrates no significant difference in all-cause mortality, bleeding events, or length of stay in the cohort of patients receiving FOBT testing. In patients undergoing endoscopy following a positive FOBT, there was no significant difference in mortality. We conclude there is currently no compelling evidence for FOBT prior to cardiac catheterization in patients hospitalized with NSTEMI.
